# LEARN 2 MOVE 7-12 years: a randomized controlled trial on the effects of a physical activity stimulation program in children with cerebral palsy

**DOI:** 10.1186/1471-2431-10-77

**Published:** 2010-11-02

**Authors:** Leontien Van Wely, Jules G Becher, Heleen A Reinders-Messelink, Eline Lindeman, Olaf Verschuren, Johannes Verheijden, Annet J Dallmeijer

**Affiliations:** 1 Department of Rehabilitation Medicine, EMGO+ Institute for Health and Care Research, Research Institute MOVE, VU University Medical Center, Amsterdam, The Netherlands; 2 Rehabilitation Center 'Revalidatie Friesland', Beetsterzwaag, The Netherlands; 3 Department of Rehabilitation Medicine, University Medical Center Groningen, The Netherlands; 4 Rudolf Magnus Institute of Neuroscience, Department of Rehabilitation, Nursing Science and Sports, University Medical Center Utrecht, The Netherlands; 5 Rehabilitation Center 'De Hoogstraat', Utrecht, The Netherlands; 6 BOSK, Association of Physically Disabled Persons and their Parents, Utrecht, The Netherlands

## Abstract

**Background:**

Regular participation in physical activities is important for all children to stay fit and healthy. Children with cerebral palsy have reduced levels of physical activity, compared to typically developing children. The aim of the LEARN 2 MOVE 7-12 study is to improve physical activity by means of a physical activity stimulation program, consisting of a lifestyle intervention and a fitness training program.

**Methods/Design:**

This study will be a 6-month single-blinded randomized controlled trial with a 6-month follow up. Fifty children with spastic cerebral palsy, aged 7 to 12 years, with Gross Motor Function Classification System levels I-III, will be recruited in pediatric physiotherapy practices and special schools for children with disabilities. The children will be randomly assigned to either the intervention group or control group. The children in the control group will continue with their regular pediatric physiotherapy, and the children in the intervention group will participate in a 6-month physical activity stimulation program. The physical activity stimulation program consists of a 6-month lifestyle intervention, in combination with a 4-month fitness training program. The lifestyle intervention includes counseling the child and the parents to adopt an active lifestyle through Motivational Interviewing, and home-based physiotherapy to practise mobility-related activities in the daily situation. Data will be collected just before the start of the intervention (T0), after the 4-month fitness training program (T4), after the 6-month lifestyle intervention (T6), and after six months of follow-up (T12). Primary outcomes are physical activity, measured with the StepWatch Activity Monitor and with self-reports. Secondary outcomes are fitness, capacity of mobility, social participation and health-related quality of life. A random coefficient analysis will be performed to determine differences in treatment effect between the control group and the intervention group, with primary outcomes and secondary outcomes as the dependent variables.

**Discussion:**

This is the first study that investigates the effect of a combined lifestyle intervention and fitness training on physical activity. Temporary effects of the fitness training are expected to be maintained by changes to an active lifestyle in daily life and in the home situation.

**Trial registration:**

This study is registered in the Dutch Trial Register as NTR2099.

## Background

Regular participation in physical activities is important for all children to stay fit and healthy. This is especially important for children with disabilities, who are often restricted in their activity options due to mobility problems. Cerebral palsy (CP) is the most common cause of physical disability in pediatric rehabilitation medicine and describes a group of disorders of the development of movement and posture, causing activity limitation, that are attributed to non-progressive disturbances that occurred in the developing fetal or infant brain[1]. Despite the non-progressive character of CP some children deteriorate in mobility related activities during childhood[2]. Current insights suggest that interventions for school-age children with CP should focus more on promoting an active lifestyle and increasing physical fitness[3]. This is the starting point of the LEARN 2 MOVE 7-12 study, that will evaluate a physical activity stimulation program in children with spastic CP aged 7 to 12 years. The LEARN 2 MOVE 7-12 study is part of the Dutch national LEARN 2 MOVE research program[4-6].

Children with CP have reduced levels of physical activity[7] and fitness[8-10] compared to typically developing children. A decrease in the level of fitness may reduce physical activity in a child with CP, and vice versa, which may result in a downward spiral of loss of muscle strength, reduced fitness and mobility, and in the long term, secondary complications due to inactivity, such as fatigue, pain, overweight, and osteoporosis[11]. This downward spiral should be broken in order to maintain current and future physical activity. This is especially important for young children, since achieving an active lifestyle between 9 and 18 years of age improves their prospects for an active lifestyle during adulthood[12].

Recent studies have shown that fitness training can improve physical fitness in children with CP[13,14]. In the only study[15] in which the effect of fitness training on both fitness and physical activity was investigated, no effect was found on physical activity[15], despite increases in fitness. The lack of a lifestyle intervention might explain this lack of effect, because positive effects on physical activity have been reported in children with no disabilities who participated in physical activity interventions[16], as well as in adults with disabilities who received counseling to adopt an active lifestyle[17].

It has been reported that, in children with CP and in typically developing children, motivation[18] and parental support[18,19], as well as self-efficacy and parental physical activity[19] influence a more active lifestyle. It is therefore important that parents are also involved in the intervention. Moreover, restricted mobility has been shown to be an important factor that limits physical activity in children with CP[7]. It is expected that practising mobility-related activities in the daily environment will result in an increase in the capacity of mobility and, in turn, provide more physical activity options. Recent published work indicating the lack of transferral from the therapy setting to the home and daily life situation[20] emphasizes this need for home-based programs.

There seems to be a lack of knowledge concerning physical activity interventions that focus on all these aspects of lifestyle, fitness and mobility in children with CP. To our knowledge, the effects of such a combined intervention have not yet been studied in children with CP. Thereby, the effects of interventions have been mostly evaluated in standardized laboratory settings, instead of in daily life situations[21].

The main aim of the LEARN 2 MOVE 7-12 study is to improve physical activity in children with CP by means of a physical activity stimulation program, consisting of a lifestyle intervention and a fitness training program. A secondary aim is to investigate the effects of this physical activity stimulation program on fitness, capacity of mobility, social participation and health-related quality of life.

## Methods/Design

### Participants and recruitment

A total of 50 children, aged 7 to 12 years, with spastic CP and Gross Motor Function Classification Scale (GMFCS) level I-III, will be included in the study. Children are included if they fulfill at least one of the following three criteria: 1) they are less active than the international physical activity norm (moderately active for one or more hours per day), 2) they do not regularly participate in sports (less than three sessions per week for 20 minutes or more), 3) they have experienced problem(s) related to daily life mobility or sports. At least one of the parents must have adequate command of the Dutch language. Children are excluded if they have instable seizures, contra-indications for physical training (such as cardiac arythmia, mitochondrial defects, or hip dysplasia), behavioral problems interfering with participation in a group, or a predominant dyskinetic or atactic movement disorder. Children who have had surgery in the past six months, and botuline toxine treatment or serial casting in the past three months (or planned to take place during the intervention period) are also excluded. On enrollment, eligibility criteria will be checked by the research co-ordinater in a telephone interview.

Pediatric physiotherapy practices, special schools for children with disabilities, and services for ambulant care will be informed about the study and the inclusion criteria. If they agree to participate, they will send information about the study to potential participants and parents. Additionally, pediatric physiatrists and the Dutch Association of Physically Disabled Persons and their Parents (BOSK) will inform families about the study by means of a brochure. All participating parents (and children who are 12 years of age and over) must sign and return the informed consent form indicating voluntary participation in the study.

### Design

This study will be a 6-month single-blinded randomized controlled trial (RCT) with a 6-month follow up, that will be performed in special schools for children with disabilities and pediatric physiotherapy practices in the Netherlands, between September 2009 and February 2012. Within each school or practice, participants will be randomly assigned to either the intervention group or the control group. A blinded independent researcher will provide the allocation sequence in sealed envelopes. For schools and practices with five or more participants, children will be stratified for GMFCS level (I *vs*. II/III) before randomization. Group allocation will only be revealed to the parents after the baseline measurements. The assessors are blinded for group allocation during the entire study period. The children and parents will be instructed not to tell the assessors which group they are assigned to. The study protocol has been approved by the Medical Ethics Committee of the VU University Medical Center in Amsterdam.

### Procedure

**Figure 1:**
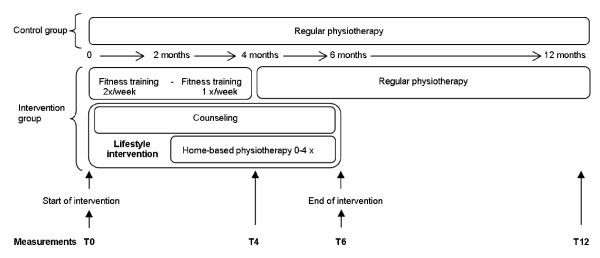
**Design of the LEARN 2 MOVE 7-12 study (intervention and measurements)**.

Children in the control group will continue with their regular pediatric physiotherapy. Since the content and frequency of the regular pediatric physiotherapy may differ among children, the physiotherapists will keep diaries about the treatment goals, and the intensity and frequency of physiotherapy sessions. Additionally, the content of the regular pediatric physiotherapy will be objectively determined by means of video-taping at least one physiotherapy session of each child. The children in the intervention group will participate in a 6-month physical activity stimulation program, instead of their regular pediatric physiotherapy. This program consists of: 1) a lifestyle intervention, combined with, 2) a fitness training program (see Figure 1).

The data will be collected during a period of one year: just before start of the intervention (T0), after the 4-month fitness training program (T4; limited measures), after the 6-month lifestyle intervention (T6), and after six months of follow-up (T12). The primary outcomes are physical activity, measured with the StepWatch activity monitor, and self-reported physical activity. Secondary outcomes are fitness, capacity of mobility, social participation and health-related quality of life.

To describe the study population the disease and environmental characteristics of the child will be measured. Disease characteristics are determined according to the GMFCS level, the Functional Mobility Scale (FMS)[22], the Manual Ability Classification System [MACS])[23], unilateral or bilateral involvement, and selective motor control of the lower extremities[24]. Environmental characteristics include type of school, living environment (rural or urban), family structure, socio-economic status, parental stress and support[25,26], and parental physical activity.

### Physical activity stimulation program

The physical activity stimulation program consists of a 6-month tailored lifestyle intervention and a 4-month fitness training program. The fitness training will replace the regular pediatric physiotherapy aimed at improving fitness or functional mobility. After the fitness training program, regular pediatric physiotherapy will be resumed during the last two months of the lifestyle intervention (see Figure 1). When there is a need for additional physiotherapy, aimed at maintaining range of motion or muscle length, this will be continued during the fitness training program.

#### Lifestyle intervention

The purpose of the lifestyle intervention is to maintain or increase physical activity and to initiate a shift towards a more active lifestyle. To that end, a 6-month lifestyle intervention has been developed, consisting of: 1) counseling children and parents to adopt an active lifestyle through Motivational Interviewing[27], and 2) home-based physiotherapy to practise mobility-related activities in the daily situation. At the start of the intervention the child and the parents together will be visited at home for a first counseling session, during which they will also be interviewed about their attitudes towards sports, physical activity of the child, and any problems they experience that are related to daily life mobility or sports. The child and the parents will be asked to rate these problems on a 10-point scale, resulting in a problem score. Based on the interview and the possible problem score, the content of the following counseling sessions and the home-based physiotherapy will be individually tailored.

Counseling is aimed at motivating the child and the parents to adopt a more active lifestyle. It consists of one or two home visits, parents visiting the fitness training, a sports workshop for the children, and one or two follow-up telephone calls. During the home visit and follow-up telephone calls the child and the parents will receive counseling, based on the Motivational Interviewing technique[27]. This is a directive and client-centered interview style that is intended to bring about behavioral change, like achieving a more active lifestyle. During the counseling, the interviewer will introduce some basic lifestyle themes (such as satisfaction with present lifestyle, and attitude towards a more active lifestyle), and will try to elicit statements concerning desire, ability, and reasons and need for changing the present lifestyle[27]. During this process the interviewer will not challenge resistance to change, but will go along with the resistance and will explore the ambivalence of the child or the parents with regard to a change in lifestyle. The interviewer then will structure possible ambivalence, in order to help the child and the parents to prioritize arguments for changing ('change talk') or not changing. When there is sufficient motivation for change, the interviewer will change the focus of the counseling to reinforce 'change talk' and to help the child and the parent to set specific goals for achieving an active lifestyle.

Home-based physiotherapy is aimed at increasing the capacity of daily activities in the home situation, in the playground or at school. The child will receive training in certain daily activities, based on the mobility problems indicated by the child and/or the parents (such as getting into the car, going up stairs carrying something, and skating). The physiotherapist will make a maximum of four visits, starting when the frequency of the fitness training has decreased to once a week (see Figure 1).

Whether or not the lifestyle intervention has been successful, will be evaluated only in the intervention group by means of the problem score and the stages of change according to Prochaska et al.[28].

#### Fitness training program

The fitness training program is aimed at increasing muscle strength and cardiovascular fitness. To increase muscle strength, the focus will be on the lower extremity extensor muscles, because the lower extremities are mainly used to perform mobility-related activities in ambulatory children with CP. It has recently been reported that children with CP can improve the strength of their lower extremity extensor muscles by means of a 12-week functional progressive resistance exercise program[29]. Cardiovascular fitness can be improved in children, by training both aerobic and anaerobic capacity. Recently, Verschuren et al.[30] reported a relationship between gross motor functioning and cardiovascular fitness in children with CP. They found a moderate to strong relationship between gross motor functioning and anaerobic capacity, but no relationship between gross motor functioning and aerobic capacity. Therefore, training anaerobic capacity seems to be most relevant for our study population. Moreover, in a recently published paper it was reported that children with CP can improve their anaerobic capacity by means of a 4-month anaerobic training program[14]. The results of that study also indicated that anaerobic training can improve aerobic capacity as well as functional muscle strength. This is in accordance with the results of a study of typically developing children, that showed an increase in aerobic capacity after anaerobic training[31].

**Figure 2:**
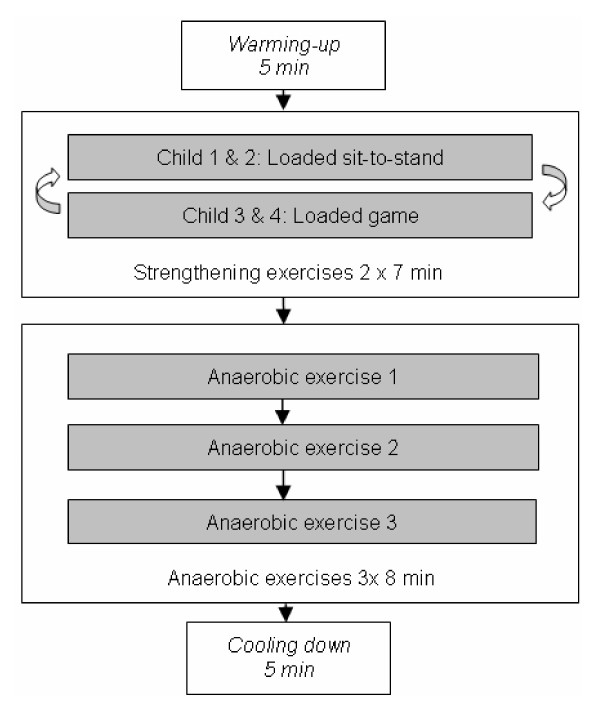
**A LEARN 2 MOVE 7-12 fitness training session**.

To achieve optimal compliance with training, it is important that the children enjoy the training. The fitness training will therefore be performed in groups of two to five children and most of the exercises will be the form of in games. The fitness training will last for four months: twice a week in the first two months, and then once a week in the following two months. The frequency will be reduced to once a week to provide the opportunity to start participating in other physical activities, such as training in a sports club. Each training session will last for one hour, starting with a 5-minute warming-up period. This will be followed by the core part of the training, consisting of two muscle strengthening exercises and three anaerobic exercises. Each training session will end with a cooling down period. Figure 2 presents an overview of a training session. The children wear their usual orthoses or orthopedic shoes during the training sessions. All the physiotherapists will have to attend workshops in which they receive instructions about the fitness training.

Lower-extremity muscle strength will be trained in a functional way with weight vests, based on the progressive strength training protocol developed by Scholtes et al.[32]. In line with the current guidelines for muscle strength training in children[33], the training load is set at three sets of 12 repetitions at the 12-repetition maximum (12RM), and will be gradually increased from only bodyweight to 100% of the 12RM. Predicted values of the 12RM, based on GMFCS level and bodyweight, are used to determine the initial training load (predicted 12RM for GMFCS I: 26% bodyweight; GMFCS II: 20% bodyweight; GMFCS III: 17% bodyweight) based on previous data[29]. The children will perform two functional strengthening exercises during each training session: 1) a *loaded sit-to-stand*, and 2) a *loaded game*. These exercises have been chosen because they involve the large muscle groups of the lower extremities which are needed for several functional mobility skills, such as rising from a chair, rising from the ground, climbing stairs, running, and stepping over obstacles.

*The loaded sit-to-stand (STS) *will be performed on a height-adjustable chair, starting with hips and knees in 90 degrees of flexion. The children have to rise from the chair in two-three seconds, and sit down again in two-three seconds, wearing a weight vest in which 'soft lead' can be placed to increase the training load. In the first week of the training the children will perform one to three sets of 12 repetitions of the loaded STS with no load (bodyweight) to get used to the exercise and the weight vest. In the second week of the training they will perform three sets of 12 repetitions with a 60-second rest interval, with a load of 40% of the predicted 12RM. When two sets of 12 repetitions are performed adequately (with fluent speed, without moving the trunk forward rapidly to initiate the movement, and without falling down on the chair seat), the children will be encouraged to perform 15 repetitions in the third set. If adequately performed, the training load will then be increased with 10-20% of the predicted 12RM. This process will be repeated during each training session (see Table 1). Since the loaded STS will be performed with a high load, this exercise is not integrated in a game.

**Table 1:** Progressively building up training load of the loaded sit-to-stand

Week	Goal	Sets	Repetitions	Load	Rest
**1**	Familiarization, focus on adequate performance	1-3	12	Bodyweight	60s

**2**	Familiarization with weight vest, focus on adequate performance	1-3	12	40% pred12RM	60s

**3**	Built up training intensity	1-2 3	12 12-15 (max)^1^	+10-20% pred12RM^1^	60s

**4-16**	Increase Strength	1-2 3	12 12-15 (max)^1^	+10-20% pred12RM^1^	60s

Abbreviation: Pred12RM = predicted 12 repetition maximum.

^1 ^If 15 repetitions are adequately performed, load is increased with 10-20% of the predicted 12RM.

*The loaded game *can vary, depending on the GMFCS level, and consists of: a) the STS, b) the forward step-up, c) the lateral step-up, or d) the half-knee rise. The training load will be set at three sets of 12 repetitions with 25% of the training load used in the loaded STS. In the first week the loaded game will be performed with only bodyweight for three sets of 12 repetitions with a 60-second rest interval. In the first three weeks the load will be increased to 25% of the predicted 12RM, and will be further increased with the loaded STS increase (25% of the load during the loaded STS). Children who are initially unable to accomplish three sets of 12 repetitions adequately, will perform the strengthening exercises with less repetitions per set and/or less sets at bodyweight until three sets of 12 repetitions will be achieved. The load will only be increased when three sets of 12 repetitions are performed adequately. The loaded game can be integrated in a game.

Anaerobic capacity will be trained the form of in games by means of three task-specific exercises (such as running or playing with a ball), that last for 15-20 seconds, each performed at maximal intensity. The training protocol developed by Verschuren et al.[14] serves as a basis for the anaerobic fitness training. To achieve a progressive work-load, the duration of the exercise will be increased and/or the rest periods will be decreased during the training period (see Table 2). In between the exercises, the children will have an active rest period (not sitting down), varying from 60 to 80 seconds, as determined by the work:rest ratio. The work:rest ratio starts at 1:5 in the first week, decreases to 1:4 in the second week, and to 1:3 in the 15^th ^week. The physiotherapists are allowed to vary the exercises or make up new exercises, as long as the children will perform the exercises at maximal intensity according to the prescribed work:rest ratio.

**Table 2:** Progressively increasing training intensity in anaerobic exercises

Week	Goal	Sets^1^	Exercise duration	Intensity	Rest	Work:rest ratio
**1**	Familiarization with the exercises	5	15 s	95-100% HRmax	75 s	1:5

**2-3**	Built up training volume by decreasing work:rest ratio	5	15 s	95-100% HRmax	60 s	1:4

**4-6**	Built up training volume by increasing exercise duration	5	20 s	95-100% HRmax	80 s	1:4

**7-8**	Built up training volume by decreasing work:rest ratio.	5	20 s	95-100% HRmax	60 s	1:3

**9-16**	Maintain anaerobic capacity	5	20 s	95-100% HRmax	60 s	1:3

Abbreviation: HRmax = maximum heart rate

^1 ^In the anaerobic exercises, a set is a 15-20 seconds exercise. Children repeat each exercise 5 times, with a varying period of rest.

To collect information about the intensity of each training session, one child per training session will wear a heart-rate monitor that can store data and collects mean heart-rate data over 5-second intervals. The physiotherapists will fill in diary logs for each child to monitor their compliance with the training and the actual content of the fitness training. They will also register any adverse effects of the training, such as muscle soreness and other complaints about musculoskeletal pain. The range of motion and spasticity of the adductors, hamstrings, soleus and gastrocnemius muscles will be measured with the Spasticity Test (SPAT)[34] to evaluate any adverse effects of the training.

### Outcome measures

The primary outcome of this study is physical activity. Physical activity will be measured objectively with the StepWatch™Activity Monitor 3.0 (StepWatch) (Cyma Corporation Seattle WA, USA) as well as subjectively with self-reports: the Activity Questionnaire for Adults and Adolescents (AQuAA), and the Children's Assessment of Participation and Enjoyment (CAPE).

*The StepWatch *is an ankle-worn bi-axial accelerometer (frontal-sagittal plane) that registers the number of steps per minute. The StepWatch can accurately record steps for different gait styles, because the sensitivity settings are calibrated for each child individually. Before each registration period, the sensitivity settings will be calibrated by comparing manual counts of a 50-step walk with StepWatch recordings. The settings will be adjusted until an agreement of > 95% is reached between manual counting and the StepWatch registration. Children will wear the StepWatch for seven consecutive days during all waking hours, except when swimming and bathing. During the registration period, the parents will fill in a weekly diary to register the child's daily activities and the weather conditions. The StepWatch is valid for children with CP, and able to discriminate between activity levels of children with different GMFCS levels[7].

*The AQuAA *is a reliable questionnaire to assess physical activity as well as sedentary behavior[35] in adolescents and adults. It is derived from the valid and reliable Dutch Short Questionnaire to Assess Health-Enhancing Physical Activity[36], but questions about sedentary behaviours were added and the recall period was specified as "the past seven days"[35]. The parents will have to rate how many days, and how much time per day the child spent on transportation to and from school, activities at school and at home, as well as leisure time activities and sports in the past seven days. The parents will also have to indicate how strenuous (light/moderate/vigorous) these activities were. For the purpose of the present study the examples of activities will be adapted, so that they are relevant for school-aged children.

*The CAPE *is a 55-item questionnaire that will be used to assess the frequency of participation in activities outside school hours[37]. The parents will be interviewed to rate the child's frequency of participation in the past four months in five types of activities (recreational, active physical, social, skill-based, and self-improvement activities). The frequency, scored on a 7-point scale, provides: a) the overall participation scores, b) the domain scores for formal and informal activities, and c) the scores for participation in each type of activity. The Dutch version of the CAPE is a valid and reliable questionnaire with which to assess the participation of children with disabilities aged 6-21 years[38].

Secondary outcomes are fitness, capacity of mobility, social participation, and health-related quality of life. Fitness is defined in this study as isometric muscle strength, aerobic capacity, anaerobic capacity, and anthropometry. Capacity of mobility is defined as gross motor functioning, functional muscle strength, and walking capacity. Health-related quality of life is as CP-related quality of life, self-reported fatigue, self-perception, and attitude towards physical activity.

*Isometric muscle strength *of the knee extensors and hip abductors will be assessed with a hand-held dynamometer (HHD)[39] (MicroFet, Biometrics, Almere) using the "make-method". The assessor stabilizes the dynamometer at the limb while the child pushes as hard as possible against the dynamometer for three seconds. Peak strength (N) is then read from the dynamometer, and the distance from the knee and hip joint to the dynamometer is measured to determine the lever arm. Moment is then calculated from peak strength and lever arm (Nm). After one practise trial, the children will perform three test trials. The means of the peak moment (Nm) over three trials will be used for the analysis. The testing positions and stabilization have been described in detail elsewhere[32].

*Aerobic capacity *will be assessed during an all-out continuous progressive cycling test on a bicycle ergometer specifically adapted for children with CP (adjustable cranks, shoe fixation, pediatric saddle) (Corival V2 Lode B.V., Groningen, the Netherlands). After a 5 to 7-minute warming-up, the initial workload will be determined, based on heart-rate (105-150 beats/min). During the test the workload will be increased every minute (1-15 Watt), based on body height and GMFCS level (range: from 0-3 Watt for GMFCS III and body height < 120 cm, to 10-15 Watt for GMFCS I/II and body height >160 cm). The children have to cycle at a constant speed (50-70 rpm), and will be verbally encouraged to keep on cycling until exhaustion. The criteria for maximal exercise are: 1) heart-rate ≥ 180 beats/min, or 2) respiratory exchange ratio (RER) ≥ 1.00, and 3) subjective signs of exhaustion. Pulmonary gas-exchange will be measured with the Quark CPET system (Cosmed, Rome, Italy) and the corresponding software (PFT CPET Suite, version 9.1a, Cosmed S.r.l, Rome, Italy) to determine breath-by-breath oxygen uptake and carbondyoxide output. Heart-rate will be measured with a Cosmed heart-rate monitor (Cosmed, Rome, Italy). Maximal oxygen uptake (VO2max) will be calculated as the highest mean values over a 30-second interval, expressed per kilogram bodyweight (ml/kg/min). Peak power (Watt) is the highest power output (maintained for at least 30 seconds) that is achieved during the test.

*Anaerobic capacity *will be assessed with the 20-second Wingate anaerobic test (WAnT20) (Wingate Software V1, Lode B.V., Groningen, the Netherlands), adapted from the original 30-second test[40], on the same bicycle ergometer. During a 4-minute warming-up, the children will perform two or three 5-second sprint practise trials to determine the optimal torque for the WAnT20, by varying the braking force (Nm) between the practise trials. After the practise trials, the children will rest for three minutes before they perform the WAnT20. They will start with one minute of comfortable cycling at 60 rpm, after which they will cycle as fast as possible for 20 seconds against the constant optimal braking force. Mean power (Watt) over 20 seconds expressed per kilogram bodyweight will be calculated as an estimate of anaerobic capacity, and used for the analysis.

*Anthropometry measures *consist of height, bodyweight, and skinfold thickness. Height and bodyweight will be used to calculate the body mass index as weight in kilograms divided by height in meters squared. Triceps and subscapular skinfold of the non-dominant arm (diplegia) or non-affected side (hemiplegia)[41] will be measured with a Holtain skinfold caliper (accuracy 0.2 mm), according to the protocol of Tanner et al.[42]. The mean skinfold thickness over three measurements per site will be used for the analysis.

*Gross motor functioning *will be evaluated with the Gross Motor Function Measure 66-item set (GMFM-66-IS)[43] which is a validated and shortened version of the GMFM-66[44]. To determine the GMFM-66-IS, the child is observed in a standardized environment, and gross motor functioning is rated on a 4-point scale by a trained assessor. The Gross Motor Ability Estimator (GMAE) software will be used to calculate the GMFM-66 interval scores (ranging from 0-100).

*Functional muscle strength *of the large muscle groups of the (most) affected leg will be evaluated by two functional exercises[45]: the 30-second lateral step-up test, and the 30-second STS test. The children will be instructed to perform as many step-ups or STSs as possible during a period of 30 seconds. If necessary, balance support will be provided. The number of repetitions performed during each test will be used for the analysis.

*Walking capacity *will be evaluated with the 1-minute walk test (1MWT)[46] on a flat, non-slippery 51-meter circular walking track. Each meter is marked with adhesive tape to make it easy to calculate the completed distance. The child will be instructed to walk for one minute as fast as possible, without running. After one minute the meter nearest to the child's position will be recorded and the total distance completed will be used for the analysis.

*Social participation *will be assessed with the Life-Habits for children[47]. Six domains of participation (fitness, personal care, housing, mobility, education, and recreation) will be assessed by means of an interview with one of the parents. The parent will have to rate: 1) the level of difficulty the child has in performing each of 36 life habits (5-point scale), and 2) the type of assistance the child needs to perform that life habit (4-point scale).

*CP-related quality of life *will be assessed with the CP-Quality of Life questionnaire[48] as a proxy parent-report. Five domains will be measured (social well-being and acceptance, functioning, participation, emotional well-being, pain and impact of disability) resulting in a total of 53 questions. The questions start with: 'How do you think your child feels about...?', and are scored on a 9-point scale.

*Self-reported fatigue *will be assessed with the PedsQL Multidimensional Fatigue Scale[49,50]. Three domains will be assessed (general fatigue, sleep/rest fatigue, and cognitive fatigue), and each domain contains six questions that start with: 'In the past month, how much of a problem has this been for you?'. The questionnaire (5-point scale) will be completed by the child, with help from the parents if necessary.

*Self-perception * will be assessed with Harter's Self Perception Profile for Children[51], adapted for children with CP[52]. The children will be assessed on three domains (motor competence, athletic competence, and global self-worth). Each domain has eight items, consisting of two opposite statements, such as "Some children think they are strong" and "Other children think they are not so strong". The children will be asked in an interview to choose which child they resemble most, and to indicate whether they are somewhat similar or very similar.

*Attitude towards physical activity *will be assessed in both the children and the parents by means of self-report. The children will have to indicate to what extent they agree with each of 14 statements, previously used in an intervention to improve physical activity in typically developing children[53]. The statements reflect eight advantages and six disadvantages of sport, for example "If I do sports, I have fun". Each statement is scored on a 5-point scale. The parents will have to indicate their opinion with regard to sports, and to what extent they agree with each of seven statements related to the accessibility of sports clubs, for example "I think my child is not good enough to join a sports club". Each statement is scored on a 5-point scale.

### Statistical analysis

A sample-size calculation revealed that at least 22 children in each group are required to detect a difference in improvement of 1,000 steps per day between the intervention and the control group on the primary outcome. The power was 0.8, and the alpha was set at 0.05. This increase in steps per day seems to be clinically relevant, since improved health outcomes have been reported in adults after an increase of 1,000 in the amount of steps per day[54]. Taking into account drop-outs, 25 children will be included in each group.

Student *t*-tests will be performed to evaluate group differences at baseline, and a random coefficient analysis[55] will be performed to determine differences in change over time (treatment effect) between the control group and the intervention group. This method takes the dependency of children within physiotherapy practices and schools into account. An intention-to-treat analysis will be performed. The primary outcome (physical activity) and secondary outcomes (fitness, capacity of mobility, social participation, and health-related quality of life) will be the dependent variables in the analysis, with group allocation (control group or intervention group) and measurement occasion (time) as independent variables. The intervention effect will be evaluated by the group * time interaction. The alpha is set at 0.05. The influence of age, gender, and disease and environmental characteristics on the treatment effect will be investigated, and, if necessary, included as covariates in the analysis. The data will be analyzed with the Statistical Package for the Social Science, version 15.0 (SPSS Inc, Chicago, Illinois, USA).

## Discussion

This is the first study of children with CP that investigates the effects of a combined intervention on physical activity, by focusing on a change in lifestyle and improvement in fitness. The separate elements of the intervention (lifestyle intervention [counseling and home-based physiotherapy] and fitness training) are expected to reinforce improvements in fitness and physical activity, resulting in sustained effects on physical activity one year after the start of the intervention. Temporary effects of the fitness training are expected to be maintained by changes to an active lifestyle in daily life and in the home situation. In turn, children may be able to maintain an active lifestyle if they have better levels of fitness and continue to perform more physical activities in their own environment.

A possible limitation of the study is that effects cannot be assigned to a specific element of the intervention. However, by assessing both physical activity and fitness outcomes, as well as the attitude of the child and the parents towards physical activity, more insight can be gained in the inter-relationship of these outcomes.

## Competing interests

The authors declare that they have no competing interests.

## Authors' contributions

LW, JGB and AJD contributed equally to this work: they participated in the design, developed the activity stimulation program, participated in the co-ordination, and drafted the manuscript. HAR, EL, OV and JV also contributed equally to this work: they advised in the developing of the study protocol. All authors participated in the reviewing process and approved the final manuscript.

## Pre-publication history

The pre-publication history for this paper can be accessed here:

http://www.biomedcentral.com/1471-2431/10/77/prepub
